# Experiences and perspectives by family caregivers on a palliative care journey: A case report from India

**DOI:** 10.1177/26323524251355286

**Published:** 2025-07-28

**Authors:** Soumya Liz Jacob, Malathi G. Nayak, Linu Sara George, Leah Macaden, Prathibha Lydia Braggs

**Affiliations:** 1 Department of Community Health Nursing, Manipal College of Nursing, Manipal Academy of Higher Education (MAHE), Karnataka, India; 2 Department of Fundamentals of Nursing, Manipal College of Nursing, Manipal Academy of Higher Education (MAHE), Karnataka, India; 3 Nursing Studies, School of Health in Social Science, The University of Edinburgh, UK; 4 Department of Mental Health Nursing, Manipal College of Nursing, Manipal Academy of Higher Education (MAHE), Karnataka, India

**Keywords:** palliative care, India, family caregivers, perspectives, focused ethnography, case report

## Abstract

Palliative care plays a vital role in supporting individuals with terminal illnesses, yet its integration and acceptance in Indian society confront significant challenges. This is despite the fact that 5.4 million people in India require palliative care annually, and <2% receive the same. Understanding the palliative care journey from the caregivers’ perspective is particularly important in the Indian context, as caregivers play a central role from diagnosis to end-of-life care and beyond. This study explores the impact of caregiving on family members of terminally ill patients in India, examining the physical, emotional, psychological, and social challenges they encounter. It unfolds the coping mechanisms and the resilience they develop throughout their caregiving journey while providing insight into their experiences, perceptions, and the complexities of their decision to choose palliative care. The study utilizes a focused ethnographic approach, collecting data from the caregivers of an older gentleman who was diagnosed with terminal alveolar cancer and metastasis along with multimorbidity through three unstructured interviews at different periods of the illness trajectory, coupled with participant observation and field notes. While highlighting caregivers' various stressors, the findings indicate that access to palliative care led to benefits such as alleviating physical burden, professional support, social inclusion, and preparation for loss. However, societal reluctance and stigma toward palliative care were evident, with family caregivers feeling inadequate or a sense of failing their duties by placing their loved one in a palliative care center. Destigmatizing palliative care can foster a more supportive and understanding environment for patients and caregivers. These findings offer insights into the complexities of the caregiving process and can potentially inform future broader investigations in the region.

## Plain language summary

Understanding the palliative care journey of family caregivers in India

This research aims to explore the experiences and perspectives of family caregivers in India who are caring for a loved one with a terminal illness. The challenges caregivers face are captured as they navigate the transitions in care, especially when deciding between curative treatments and palliative care (PC). The study seeks to understand the factors influencing caregivers’ choices, the stress they endure throughout the illness, and the coping mechanisms they use. The demand for palliative care has increased globally, especially in low- and middle-income countries, due to aging populations and rising disease burdens, with COVID-19 worsening this need. Despite being a human right, palliative care remains a significant unmet need worldwide, with research showing benefits when provided early in life-limiting illnesses. An ethnographic approach was used to understand caregivers’ lived experiences in their specific cultural and social context. Interviews, observations, and document analysis helped capture the complexities of caregiving. The key participants were caregivers of Mr. Ben (pseudonym), who had a terminal illness. Mr. Ben, a 78-year-old man with a history of stroke and chronic kidney disease, was diagnosed with terminal cancer. His wife was the primary caregiver, supported by their daughter, a professional. Both caregivers were involved in community and religious activities. The caregivers of individuals with terminal illnesses in India face intense emotional, physical, and practical challenges throughout the illness journey. Access to palliative care significantly alleviates physical, emotional, and psychological burdens on caregivers. However, societal reluctance and stigma hinder its wider adoption and acceptance. This work is a valuable resource for healthcare providers, policymakers, and social workers working to improve end-of-life care.

## Introduction

The global demand for palliative care (PC) is exponentially increasing due to an aging population, rising non-communicable diseases, and the impact of COVID-19. Each year, around 56.8 million people need PC, with 78% living in low- and middle-income countries.^1^ In India, where an estimated 5.4 million people require PC <2% receive it annually, placing the country 67th out of 80 on the Quality of Death Index.^2^ The approach to PC is evolving, shifting from end-of-life care to being integrated from the point of diagnosis of a life-limiting illness.^3^ People with life-limiting illnesses often overestimate treatment benefits due to misinformation or misunderstanding of the likely outcomes of aggressive curative care.^4^ From recent death and dying discourses, dying is often prolonged, and death comes later for many.^5^ PC encompasses a philosophy of care and a system for delivering highly structured and organized care to patients with life-limiting illnesses, improving human dignity, better self-care, coping skills, and quality of life for patients and their families from diagnosis until death and bereavement.^6^ Family caregivers often perform practical tasks, provide emotional support, relieve pain and other symptoms, and communicate with health services. They usually prioritize the patient’s needs and well-being over their own.^7^ In addition to managing the patient’s worsening physical dysfunction^8^ and mounting psychological distress, family caregivers of patients with advanced cancer also deal with feelings of loss of control and anticipatory grief, which can exacerbate caregiver burden.^9^ Those with life-limiting diseases and their families are vulnerable to detrimental financial, social, and emotional outcomes, as family caregivers may exhibit various coping mechanisms and behaviors that may also impact patient well-being and family dynamics.^10^ This offers a critical opportunity to consider the role and input of family caregivers as a cornerstone of PC.

In India, caregivers have a central role across the care continuum, as caregiving is an expected duty here.^11^ Families are deeply involved in determining the course of treatment, including decisions about curative versus PC.^12^ Family members often end up providing informal care to patients due to various reasons like cultural norms, limited availability of professional care, or not having enough funds.^13^ Given the collectivist nature of Indian society, supportive tasks such as emotional support, daily care, following treatment plans, and assisting with medical decisions are shouldered by caregivers with personal challenges and costs.^14^ While the role of caregivers is socially and culturally embedded, it is not legally defined. In many cases, family members, rather than patients, make critical treatment decisions, particularly when the patient is perceived as vulnerable or unable to handle distressing information.^15^ Physicians frequently seek family consent before treatment, reinforcing the family’s authority in medical decision-making. However, this practice raises ethical considerations regarding patient autonomy and informed consent, as patients may not always be fully informed about their diagnosis or prognosis.^11^

Research has proven that chronic stress from caring for a terminally ill patient can lead to heightened inflammation and health issues like immune dysfunction, high blood pressure, and heart disease.^16^ The perspectives of caregivers during terminal illness and their experiences with the transition to PC remain underexplored in the Indian context. In this study, caregiver perspectives refer to those family members’ experiences, challenges, and emotional journeys as primary caregivers providing care to individuals with terminal illnesses. This incorporates the entire process from the diagnosis and progression of the disease to the patient’s death and subsequent bereavement. Caregiver perspectives highlight the complex dimensions of this journey, including the grieving process, as they encounter both the loss of their loved one and their life transitions without their caregiving role.

As a nurse researcher working in the field of PC, I was particularly interested in the human response to terminal illness and their experience in choosing PC over curative therapies in a society that is not so PC-affluent. The focused ethnographic approach^17^ utilized in the study is detailed here so that the readers can transparently examine the study procedure and the study’s rigor.

## Objectives

To examine the physical, emotional, psychological, and social impacts of caregiving on family members of terminally ill patients in India.

To identify the key stressors family caregivers face and the coping mechanisms they employ throughout the caregiving journey.

To explore the factors influencing family caregivers’ decisions to choose PC over curative treatment and to understand their perceptions and experiences of this decision-making process.

## Methodology

This is a case report enriched through a focused ethnographic approach^18^ that explores and describes the experience of caregivers during the PC journey of a patient, from the diagnosis of a life-limiting illness to death and bereavement. A comprehensive study of an individual or case may produce rich data establishing precedents in clinical literature.^19^ It may unveil potentially important, previously unnoticed phenomena that contribute immensely to the discipline’s body of knowledge.^20^ The reporting of this study conforms to the CARE (CAse REport) checklist provided as supplemental material. A focused ethnography was chosen, as it is particularly effective in understanding individuals’ lived experiences and perspectives within specific cultural and social contexts.^21^ This allows for examining a single issue or subject over a brief period with an eye on data collection by carefully choosing participants aware of the topic under study.^22^ Mr. Ben’s (pseudonym) caregivers were purposefully/opportunistically^23^ selected for the study. The researcher did not have a pre-existing personal relationship with the participants; however, a rapport was established through a broader project based on shared interests in the subject matter. The caregivers actively contributed their insights and experiences, expressing a keen interest in future collaborations with the researcher regarding various aspects of the disease and caregiving process. This openness and willingness to engage further led to their recruitment. Beyond their caregiving roles, the spouse volunteers at a PC center near their home, and the daughter is involved in community-based social service initiatives. The daughter resides separately with her family but maintains close involvement by visiting every other day. This regular presence ensures her active participation in caregiving despite not living in the same household.

In ethnography, researchers observe people’s daily lives, listen to their conversations, engage in formal and informal inquiries with individuals, and gather relevant documents.^17^ Reflexivity was addressed through a triangulation inquiry, aiming to critically reflect on the researcher’s role and the influence of others in shaping the knowledge produced.^24^ The core questions the researcher (first author) asked herself were:

1. How has the first author influenced what was learned?
2. How do the participants perceive themselves in the study?
3. How might the study context have influenced what the first author learned?

The researchers’ assumptions about caregiving and emotional reactions may have shaped the focus of the interviews, observation, and steered the analysis, potentially overshadowing personal and cultural dimensions. Thus, the researcher reflected on whether her expectations had shaped the data collection process or whether the caregivers’ lived realities had truly been captured. A strong researcher–participant relationship based on perceived expectations or trust was key to disclosures, especially on sensitive topics like emotional strain. Characteristics such as age, gender, and cultural background shaped how they engaged with the researcher and influenced the data collected.

India’s cultural and social context, including norms around family caregiving and stigma around terminal illness^25^ shapes caregivers’ perceptions and engagement with PC. The healthcare system’s challenges, including limited access, regional disparities, and a shortage of trained professionals, compound caregivers’ struggles to find adequate care.^26^ Additionally, political and economic factors, such as government funding, private healthcare availability, and the financial burden on families, further impact caregivers’ experiences.^2^ These contextual factors are crucial to understanding the family caregivers’ challenges and would likely differ if the study were conducted in a more developed healthcare setting.

### Case description

Mr. Ben is a 78-year-old active retired military service gentleman. He had a cerebrovascular accident (CVA) in the year 2017, diabetes, and hypertension for more than 20 years, and has been on conservative management for chronic kidney disease for the past 5 years. The CVA had left him with a right-sided weakness. He could do his daily activities with minimal assistance, mostly confined to his house. In November 2023, he presented with a submandibular node that was tender and growing to a general surgeon at a tertiary center. Investigations revealed carcinoma of the left upper alveolus with level 1b metastasis. The family then consulted with medical and surgical oncologists, who discussed active cancer treatment modalities. His wife is the primary caregiver, 78 years old, a retired employee, and his daughter is a professional. Both are involved in their community’s social and religious activities and are highly family-bound, like most people in the region.

### Data collection

Data collection happened in different settings during the transitions between tertiary centers, hospice, and home care environments. To immerse oneself in the social context of the participants, interviews and informal interactions, including telephone conversations, visits, and access to medical records, were used, which facilitated appropriate interpretations of the topic in real-time^22^ concurrently and sequentially.^27^ Written informed consent was obtained from both the caregivers for the study. Open-ended questions were asked, and the conversation was audio-recorded. Health records and participant observations with field notes supplemented the data. The first author anchored her position as a researcher by observing the situation, asking the participants what was happening, and interpreting the perspectives, thereby closing the ethnographic picture.

### Interview

Three unstructured interviews with open-ended questions that were audio-recorded explored the PC journey. Unstructured interviews have no established question or answer categories; instead, they focus on social interaction between the researcher and the participants.^28^ “Punch (1998) described unstructured interviews as a way to understand the complex behavior of people without imposing any a priori categorization, which might limit the field of inquiry.”^9^(p.1) The interview questions were not predetermined but spontaneously generated^28^ based on the participants’ narrations during the conversations. This approach allowed the researcher to keep the study’s purpose^29^ in mind to explore the viewpoints pertinent to the issues she was interested in. While the broad focus of the interviews remained on the caregiving journey, the specific focus was on (1) the initial diagnosis and early caregiving experiences, (2) challenges, decision-making, and adaptations during the transition from curative to PC, and (3) reflections on caregiving during bereavement. The questions evolved in response to participants’ accounts, allowing for a deeper exploration of their lived experiences and perspectives. A series of interviews, each lasting 30–40 min, was conducted at a convenient time and location for the participants. The first interview was with the daughter at the café (a secluded area with privacy) while Mr. Ben was receiving care at the acute care hospital. The second was with both wife and daughter at his daughter’s home during his stay at the PC center, and the third was with the wife at Mr. Ben’s home during the bereavement period. Personal encounters for observation were also conducted with the caregivers at different time points.

### Participant observation and field notes

Ethnographers study people’s daily lives using an emic (inside) and etic (outside) approach to describe communities and cultures.^30^ Participant observation allows the researcher to understand how people interrelate, identify cultural priorities and values, and build rapport with informants, thereby facilitating deeper insight and more effective research.^31^ Coupled with field notes, participant observation is considered a reliable mode of expressing findings and is also highly credible as a supplementary data source.^30^

Field notes include facts such as the physical setting, the people involved, and a complete description of the activities witnessed.^24^ At the same time, field notes also provide the outsider/researcher’s perspectives and reflections.^23^ The researcher’s observations and reflections were separately recorded. In this study, these findings only supplemented the interview data with a better understanding of the situations and concepts.

### The theoretical framework

The illness Trajectory Model by Strauss and Corbin (1992) developed as a grounded theory through extensive research on dying^32^ can reveal the dynamic perspectives of caregivers as they manage the illness of their loved one.^33^ The model underpins the fact that chronic illness has a trajectory and can be controlled through distinct sub-phases.^34^ In this study, the application of this model is limited to the course of illness as perceived by Mr. Ben’s caregivers. The framework consists of eight stages describing the complex, non-linear nature of living with chronic illness and the changes in both health and quality of life over time.^35^ The illness trajectory of the study is explained in Table 1.

**Table 1.** Application of the illness trajectory framework to Mr. Ben’s case study.

Stages	Description
1. *Pretrajectory*: Risk factors are present but illness has not yet developed	This study traces the illness trajectory beginning in November 2023, when Mr Das received a cancer diagnosis. Retrospective data gathered from caregivers provides additional context and is detailed under the section titled “Study Context and Setting” in this paper.
2. *Trajectory onset*: Marked by the appearance of symptoms and diagnosis	The onset of symptoms was first noted when Mr. Ben observed a small growth near the gum in his mouth, accompanied by mild discomfort. His spouse subsequently took him to a dentist and later to a surgeon, who prescribed antibiotics while offering reassurance that the lesion was likely benign. Despite this initial treatment, the lesion persisted without significant change over one week. In response to the ongoing symptoms, a biopsy was performed. At this stage, given the presence of pain, the family continued to suspect the lesion was due to an infection. However, the biopsy results revealed the presence of carcinoma in the left upper alveolus, with metastasis to level 1b lymph nodes.
3. *Acute stage*: Where the illness requires treatment but is somewhat controlled	He slowly started developing swelling over the left side of his face, and the mass appeared a bit more thickened, according to his spouse. In-person and proxy consultations were carried out regarding the course of treatment. He was referred to medical and surgical oncology for further evaluation, and a treatment plan was outlined and given. This plan prompted the family to carefully consider the feasibility of proceeding with the recommended extensive treatment, particularly given the patient’s advanced age and the presence of comorbid conditions. Compounding the decision-making process was the lack of medical coverage at the current healthcare facility, necessitating the family to seek care at a distant medical institution. As they navigated this complex situation, the family faced conflicting opinions regarding the most appropriate action. Meanwhile, the condition appeared to deteriorate, with the growth visibly enlarging and Mr. Ben presenting a progressive decline in his health daily.
4. *Crisis*: A period of acute illness or exacerbation requiring urgent care	To better understand the situation and reach a consensus regarding the appropriate treatment approach, the family sought the advice of their PCP, who facilitated a consultation with a PCP. During this consultation, the family was provided with a clear and comprehensive prognosis, including options for both curative and PC. The PCP recommended an additional meeting with the multidisciplinary oncology and PC teams to discuss treatment options with the family further. At this meeting, the cancer diagnosis was formally communicated to Mr. Ben, and counseling was provided through a family-centered approach, ensuring transparency and avoiding any form of collusion. “He preferred not to have any deep treatment, but some superficial ones so that he could be happy till his end.” Reports his daughter. At this juncture, the primary caregiver (wife) found herself grappling with conflicting emotions about whether to prioritize PC or pursue aggressive curative treatment. This decision was particularly challenging given Mr. Ben's expressed wishes and the emotional and practical considerations surrounding his care. Within one week, the family wholeheartedly and unanimously chose PC as a way forward.
5. *Stable*: When the illness is under control, and the person functions at a baseline level	Mr. Ben remained at home, adhering to his routine with supportive care. His mood notably improved after the primary caregiver brought a pet cat from a nearby town, which enhanced his emotional well-being. The radiation oncologist developed a care plan, and referrals were made to a local facility for palliative radiation therapy. The treatment, consisting of five sessions as a palliative dose, was initiated according to the patient’s tolerance. The primary caregiver also adapted changes in diet, preparing gruels and mashed foods to facilitate easier swallowing.
6. *Unstable*: When symptoms worsen or fluctuate, necessitating intervention	Mr. Ben experienced two episodes of bleeding from the lesion site, which was successfully managed with low-dose radiation therapy despite recommendations from the emergency department for vascular surgery. According to Mr Ben’s daughter, “It was not easy for him for a day or two after radiation. He used to be very tired and moody; later, he used to pick up.” Over time, a gradual deterioration in the patient’s overall health status was observed. This included exacerbating preexisting symptoms such as confusion and decreased muscle strength. In addition, several new concerns emerged, including restricted mobility, urinary retention, difficulty awakening, loss of appetite, occasional vomiting, and the development of skin breakdown.
7. *Downward*: Where there is a progressive decline in health and function	As the disease progressed, care at home became increasingly difficult. In July 2024, he presented with an episode of vomiting followed by shivering and high-grade fever. He was then shifted to a tertiary center, where he was admitted and stabilized in the ICU. They insisted on starting hemodialysis and even got venous access for the same. The family, now used to the baseline parameters, found no alarming variation requiring active intervention. They shifted him to a Palliative Center after consenting to the facility against their advice for aggressive management. The PC center took over his care and significantly reduced the caregiver burden.
8. *Dying*: The final stage of illness, marked by the approach to death	Mr. Ben’s health deteriorated gradually, and the family noted the increasing signs of decline. The daughter reflected, “The absence of dad at home helped my mother prepare mentally for his permanent departure.” Surrounded by family, he passed away peacefully on August 15, coinciding with India’s Independence Day, a day that held special significance for him as a former military serviceman. The daughter recalled a poignant moment when Mr. Ben’s primary nurse from the Palliative Center, visited their home on the day of the funeral to pay a personal tribute to Mr. Ben. The family described this act as a testament to the profoundly compassionate nature of PC, recognizing that such care indeed stems from the providers’ hearts. The family hosted a condolence gathering in gratitude and respect at the PC center. The primary caregiver has continued to maintain a connection with the center, accentuating the lasting impact of the care received.

PC: palliative care; PCP: palliative care physician.

### Qualitative analytic procedure

The audio-recorded qualitative data were transcribed and read repeatedly to ensure familiarization. Line-by-line coding using an inductive approach was done using Open Code software 4.0. Themes were identified and rechecked with the codes and verbatim for coherence. Member checking was done by taking the coded data back to the participants to ensure that the data was an accurate representation of their perception.^24,36^ This process helped to enhance rigor in the qualitative data.^37^ A second reviewer independently reviewed, validated, and revised themes. Consensus was generated by mutual discussions on the biases, considering the frequency of occurrence in the transcripts and the interpretation, bringing in the investigator’s triangulation.^24^ The themes were then finalized and defined, followed by the generation of the report.

## Results

Using a thematic analysis approach,^38^ several significant themes surfaced that shed light on their physical, psychological, social, and emotional journey. These revelations offer a clear picture of caregivers’ difficulties and resiliencies at this stage of life. The data were collated and analyzed to develop collective themes and insights into the entire PC journey, represented in Table 2. The verbatim quotes by the wife (denoted as **S**) and the daughter (denoted as **D**) are the basis for the analysis.

**Table 2.** Themes and insights from qualitative data.

Illness trajectory phase	Verbatim quotes	Caregiver perceptions and experiences	Thematic insight
Onset	“Just the day before this happened, he was up on a tree and doing things at home” (D). “Even the treating doctor said that since the node was painful, there was nothing to worry about. To rule out biopsy was done, but we never expected” (S). “Dad was very meticulous in everything and a very jovial person. No clue of how he ended up with all these” (D). “Dad used to ask me when he could climb trees again. I had to pacify him, for I knew it would not happen again” (D).	Initial shock, confusion, disbelief	Unanticipated health status and emotional turmoil
Acute phase	“One morning I found some blood stains on his pillow cover. When I shone a torch, I could see flesh-like thing bleeding from his mouth. I felt something very unusual” (S). “Telling right on our face that his condition is bad was not the right way. Although we knew what his diagnosis could take us to, it hit us pretty hard” (S). “Undergoing surgery and chemotherapy and radiation at this point may not be possible. He is already old and weak” (S). “The doctor (PC physician) addressed him as uncle ‘X’ and explained his disease condition calmly and gave him choices for treatment” (D). “Since he had Central Government Health Scheme (CGHS), we found that certain services are not covered in the scheme, which would cost us a lot. So, we had to change hospital” (S).	Emotional distress, treatment decisions, hospitalizations	Crisis and complex decision-making
Crisis phase	“Dad almost sleeps all day. He is dependent for all his needs” (D). “Now mummy has to take complete care of Dad. Toileting, bathing, feeding, and everything. Managing a household is another big task” (D). “Now mummy took over giving him medicines. Before, she knew nothing about his medications” (D). “Mamma was not much in favor of the idea of PC. You know she has worked as a volunteer there. So she knows what happens at the end” (D). “We had to give in writing to the hospital that no more treatment is needed so that we could shift him to hospice. It wasn’t just easy, you know” (D). “I had to strictly tell them that we do not need dialysis for Dad at this stage. The doctor was telling us that we could prolong his life for a few more months. I thought it was meaningless to give him that much strain” (D).	Heightened emotional strain, decision-making under pressure, dependence	Increased physical, emotional, and practical burdens
Stable	“I got some nice Calicut biryani, which Mom and Dad like, and to our surprise, Dad had it well” (D). “I think he is much better after coming home. At least he has his things and the comfort of his bed, you know” (S). “Mummy got a Persian kitten, which keeps Dad engaged. We can see a significant elevation in his mood” (D). “I was surprised when he addressed me ‘GRACE’ something he had forgotten for a long time” (S).	Emotional relief and fulfillment, sense of empowerment, alleviation of stress	Restoration of some normalcy
Unstable	“It was frightening to see so much blood in his mouth. I didn’t know what to do” (S). “They managed somehow temporarily but told us that some big thing should be done to stop the bleeding, which is quite costly also” (D). “Without calling mummy, Dad got up from the bed and fell. It was tough to put him back to bed” (D).	Fear and uncertainty, encountering new physical challenges	Adversity with new challenges
Downward phase	“He takes a long time to swallow. I use a mixer to make his food into easily swallowable form” (S). “He almost lost control over passing stool. It is very watery, and he is unwilling to use pampers. Sometimes I find him wet. It is difficult” (S). “I am also torn between my academic responsibilities, children, and dad’s sickness” (D).	Complex caregiving routines, ongoing adjustments, exhaustion	Feeling of helplessness
Dying phase	“We made some structural changes at home to shift Dad easily” (D). “I think our system is still focusing only on the treatment part. We had to turn them down to shift to the hospice, literally. It is painful to see with the catheter inserted in his neck” (D).	Focus on comfort, dignity, acceptance	Managing family and societal expectations Community support Transition to a new normal
	“My brother was arguing with mummy when dad was deteriorating.” “How can you just leave him like that? He asked. Mummy had to sit down and explain (D).	Emotional distress and conflict over treatment decisions.	
	“You should see mummy; she has lost much weight and is very tired also. But she will ensure that dad is well-kept and well-fed” (D).	Self-neglect	
	“We know that we may have to rush him to the hospital when we cannot manage at home. But people will think that since we do not want the burden, we have kept him in the Palliative center” (D). “People will think that to save money, we have put him in Palliative care” (S). “I am ready to take care of him even in a hospital general ward. It is not that we are short of money” (S). “People think that we don’t want to spend money and keep a home nurse and take care at home” (S). “They think that if we are not doing operation for Cancer and taking some other treatment, we are ready to let our person die” (D). “I know that in the Palliative Center, people will die soon. I have seen many people” (S).	Fulfilling societal norms, destigmatizing PC	
	“Many patients are fighting the same battle as ours. There was also a patient who had maggot-infested wound” (S).	Heightened awareness and emotional sensitivity	
	“Dad’s absence at home, in one way, was helping us to prepare for the time when he is not going to be with us anymore” (D). “I was there (PC center) with him throughout the day, and at night, I get home and sleep. This is helping me a lot” (S). “In the hospice, people were coming in and interacting. We even knew some of them” (D). “Dada was looking fresh after the nice coconut oil massage and bath. He responds to video calls and appears happy” (D). “I recollect the death of my uncle while I was still a child, later my grandmother, and what we went through” (D). “Dad received Viaticum while at the hospice” (D). “All of us were with Dad, including grandchildren. He was peaceful and comfortable. The only thing we could hear was a rattle during his breathing” (D).	Grief, anticipatory grief, adjusting to life after loss, appreciating support	
	“The Hospice would preserve the body for 24 hours from the time of death” (D). “In no time, our neighbors made arrangements at home for his final journey. We just had to standstill” (D). “Members of the military men’s association paid homage during the funeral, which was attended by many” (D).	Rituals and community support in death	
Bereavement	“On the seventh day of dad’s demise, mummy held a thanksgiving at the PC center. She said there is no better place to do this than there” (D).“I used to take medicines at the same time medicines were given to him. Now that he is gone, I just forget to take mine” (S).		

**Figure 1.**
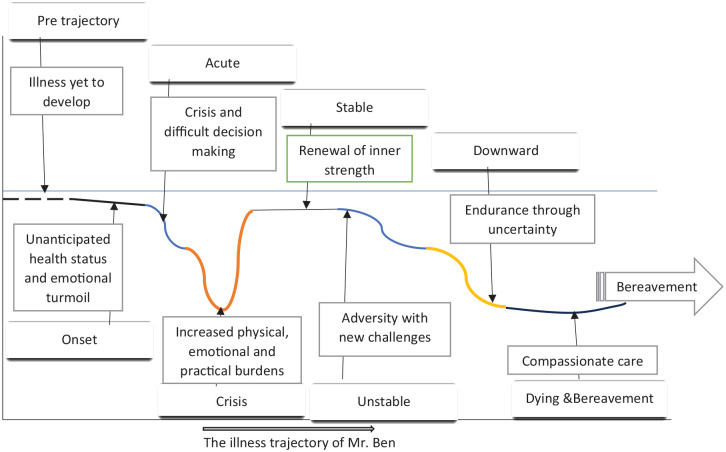
Thematic representation of the caregivers’ PC journey. PC: palliative care.

The perspectives and experiences of a caregiver, as depicted in Figure 1, take off with an emotional whirlwind when the caregiver first learns about the illness. This unanticipated health status causes confusion and disbelief. Emotional distress, hospitalizations, and complex treatment decisions mark the acute phase. The caregivers were overwhelmed by the complexity of the illness, medical options, and the uncertainty surrounding both. The crisis phase shows intense emotional strain and decision-making under pressure, mounting the caregiver with physical, emotional, and practical burdens with little or no relief, showing a steep downward curve.

A relatively more manageable caregiving experience follows this. In the stable phase, some emotional relief, empowerment, and stress reduction are noted among the caregivers. It is worth noticing that they feel fulfilled despite the tedious journey. Continuing this phase, the caregivers experience new challenges due to changing physical status. In their pursuit to cling on, they succumb to strategies to overcome their fatigue in this downward phase. The dying phase is where the caregiver is focused on providing comfort, maintaining dignity, and accepting the impending death of the loved one. In this phase, the transition to the hospice has been made. The caregivers encountered heightened emotional distress and decisions over treatment options on one side and overcoming the stigma associated with PC on the other side. It is worth noting that a certain amount of emotional distress, self-neglect, and efforts to fulfill societal norms are reflected throughout the PC journey.

## Discussion

### Implications for clinical care

Caregivers’ perspectives are mostly unnoticed or taken for granted and are an understudied topic in PC. Family caregivers in PC services are the support providers within the patient-family caregiver relationship.^39^ Caregivers experience turbulence from the time of diagnosis, during the process of caregiving, and toward the end of life and the bereavement period.^40^ In this study context, the caregivers manifested a broad spectrum of support for Mr. Ben, as evidenced in the thematic analysis. Their journey involves varying degrees of stress^41^ and adaptation. The illness trajectory framework in this study showcases the transition of caregiving phases. The dynamic nature of the disease trajectory presents varying caregiving demands underpinned by the theory.^32^ There is a significant physical and emotional strain on the primary caregiver, as confirmed by previous research.^42^ Even though the road to PC was their personal choice in this study, the family could reap the benefits of PC after accepting the disease condition and probable prognosis.^43^

### Unseen emotional and physical burdens

The emotional experience of the caregivers begins with shock and disbelief and evolves at every phase of the journey. This echoes the broader literature on the emotional impact of caregiving upon initial diagnosis of the life-limiting illness and associated uncertainties.^44^ The acute phase marked the beginning of a physical toll along with emotional exhaustion on the caregivers as they managed the demands of frequent hospitalization and crucial decision-making. The crisis phase intensifies the turmoil with the increasing complexity of the medical condition and treatment options. Here, they had to think critically and execute strategies to overcome the practical burdens of caregiving. Family caregivers exhibit extensive physical and emotional challenges while caring for their loved ones.^7^ The stable phase brings about a notable change in the trajectory, with a feeling of relief and empowerment in handling the situation. These results are consistent with earlier studies showing that caregivers gradually build resilience and coping skills that lessen the severity of the caring experience.^45^ However, given the nature of the disease, this phase was short-lived, and they were pushed into the prodigious path of worsening disease with substantial limitations on their everyday life.

The downward phase takes over with a transition through an unstable bit, reflecting the caregiver’s strain over the decline of their loved one’s health condition. There is consistent evidence that the continuing nature of caregiver experience leads to burnout, with caregivers struggling to meet the emotional and physical demands of the role.^46^ The dying phase is the most emotionally intense segment in the trajectory, where the eventual acceptance has set in, with a focus on providing comfort, maintaining dignity, and preparing for the inevitable death of their loved one. Furthermore, the stigma surrounding PC pressurized caregivers to prioritize the needs of their loved ones to the detriment of their own physical and mental well-being.

### Social and cultural implications

There are social and cultural dimensions of caregiving, particularly societal expectations, that shape caregivers’ choices, responses, and coping strategies. The caregivers view the existing cultural framework demanding caregiving as a duty, commitment, and moral obligation. They had to manage expectations, often at the expense of their well-being, and were torn between fulfilling their societal roles and their own needs, resulting in self-neglect, especially the primary caregiver (spouse) in this study. The societal norms exacerbate emotions of guilt and responsibility, evident in the crisis and dying stages, as verbalized by the primary caregiver, who was quite mindful of the transition to PC. Studies ascertain that societal perceptions influence self-perception and caregiving decisions, and in certain cultures, handing over the care of your loved one to professionals may be looked down upon as an act of neglect.^47^ Studies have emphasized the need for social support as a means of reducing emotional impact and alleviating their burdens.^48^ In this study, the support from the PC center and the familial support helped the primary caregiver balance caregiving and her own needs to some extent. Caregivers expressed gratitude during the bereavement by holding a thanksgiving service in the PC center. The wife even verbalized the idea of becoming a PC volunteer. Expressing gratitude^10^ is a form of emotion caregivers manifest when they adopt PC.^43^

## Limitations

Reliance on unstructured interviews of a single case has inherently limited the generalizability of the findings. The experiences presented are unique to the participants and may not be representative of other family caregivers in PC with different diagnoses, backgrounds, and cultural contexts. The subjective nature of individual perspectives and data collection at varying illness stages may have introduced biases and led to the omission of significant areas. Although the findings provide some insight, a more detailed investigation is needed to fully understand the factors and dynamics influencing their decision-making processes in transitions to PC. Future research could further explore this aspect to provide a more comprehensive understanding.

## Strengths

The study explores the dimensions of family caregivers’ perspectives and engagement with PC beyond surface-level aspects. This in-depth approach unveils the iceberg phenomenon of caregivers’ experiences and perspectives throughout the terminal illness trajectory, revealing dimensions other methodologies could potentially overlook.

## Conclusion

The highlight of the study is its real-life context where PC is not so affluent. It highlights the importance of integrating PC early in treatment for patients with complex conditions. Mr. Ben lived for over 13 months after diagnosis without aggressive therapy, emphasizing the need for holistic, accessible care through end-of-life. His case illustrates how palliative interventions support patients and families, emphasizing autonomy and quality of life. The family’s experiences reflect everyday struggles in terminal illness, making this a valuable example of patient-family-centered care. Cultural sensitivities in PC also emerged, calling for a shift in societal perceptions. Future research could explore PC across conditions, caregiving roles, and quality of life outcomes. It is an eye-opener to integrate PC in acute settings and facilitate the smooth transition of patients when required, where patients, significant others, and healthcare providers would benefit.

## Supplemental Material

sj-pdf-1-pcr-10.1177_26323524251355286 – Supplemental material for Experiences and perspectives by family caregivers on a palliative care journey: A case report from India Supplemental material, sj-pdf-1-pcr-10.1177_26323524251355286 for Experiences and perspectives by family caregivers on a palliative care journey: A case report from India by Soumya Liz Jacob, Malathi G. Nayak, Linu Sara George, Leah Macaden and Prathibha Lydia Braggs in Palliative Care and Social Practice
